# Investigation of the Optoelectronic Properties of Ti-doped Indium Tin Oxide Thin Film

**DOI:** 10.3390/ma8095316

**Published:** 2015-09-21

**Authors:** Nen-Wen Pu, Wei-Sheng Liu, Huai-Ming Cheng, Hung-Chun Hu, Wei-Ting Hsieh, Hau-Wei Yu, Shih-Chang Liang

**Affiliations:** 1 Department of Photonics Engineering, Yuan Ze University, Chung-Li 32003, Taiwan; E-Mails: nwpuccit@saturn.yzu.edu.tw (N.-W.P.); s1005623@mail.yzu.edu.tw (H.-M.C.); yzu70739hu@gmail.com (H.-C.H.); awxyi9967@gmail.com (W.-T.H.); s1015623@mail.yzu.edu.tw (H.-W.Y.); 2 Materials & Electro-Optics Research Division, Chung-Shan Institute of Science and Technology, Lung Tan 32599, Taiwan; E-Mail: lhyh912601@gmail.com

**Keywords:** oxide-related compound, indium tin oxide (ITO), magnetron sputtering, transparent conducting oxide (TCO)

## Abstract

In this study, direct-current magnetron sputtering was used to fabricate Ti-doped indium tin oxide (ITO) thin films. The sputtering power during the 350-nm-thick thin-film production process was fixed at 100 W with substrate temperatures increasing from room temperature to 500 °C. The Ti-doped ITO thin films exhibited superior thin-film resistivity (1.5 × 10^−4^ Ω/cm), carrier concentration (4.1 × 10^21^ cm^−3^), carrier mobility (10 cm^2^/Vs), and mean visible-light transmittance (90%) at wavelengths of 400–800 nm at a deposition temperature of 400 °C. The superior carrier concentration of the Ti-doped ITO alloys (>10^21^ cm^−3^) with a high figure of merit (81.1 × 10^−3^ Ω^−1^) demonstrate the pronounced contribution of Ti doping, indicating their high suitability for application in optoelectronic devices.

## 1. Introduction

Transparent conducting oxide (TCO) thin films can be fabricated using various types of material, such as tin oxide, indium oxide, and zinc oxide [1]. Indium tin oxide (ITO) thin film is currently the most commonly-applied TCO thin film. The low resistivity and high visible (VIS)-light transmittance of ITO thin films enable them to be applied in the production of numerous optoelectronic devices such as liquid crystal displays [2], flat panel displays [3], and touch panels [4]. ITO thin films can be fabricated using various methods such as magnetron sputtering [5], the sol-gel method [6], chemical vapor deposition [7], and pulsed laser deposition [8]. Among these methods, magnetron sputtering deposition is cost effective and suitable for producing large-area thin films. This method involves using argon ions to sputter particles onto a substrate in preparation for thin-film deposition. Through the conversion of high potential energy into kinetic energy, the particles on the substrate can be imbued with additional energy, thereby facilitating crystallite nucleation, which can improve the optoelectronic properties of thin films. Furthermore, magnetron sputtering enables production at low substrate temperatures, and can be combined with other methods that improve optoelectronic properties to produce ITO thin films. These methods include substrate-heating during the thin-film deposition [9], and post-growth thermal annealing [10], which can effectively improve the crystallinity and optoelectronic properties of TCO thin films. During the fabrication of TCO films with high carrier mobility, transition-metal elements, such as Ti, Mo, W and Nb, are typically doped into indium oxide (In_2_O_3_), improving their optoelectronic characteristics considerably [11,12,13,14].

In_2_O_3_ is mixed with these transition metals because the ion radii of these metals are smaller than that of indium oxide [15]; therefore, using transition metals as dopants does not substantially alter the crystal structure of indium oxide. Moreover, the strength of the Lewis acid in these transition metals is higher than that of indium oxide-based semiconductors; thus, transition metals easily release free electrons, thereby increasing the thin-film conductivity and improving the carrier mobility because of the reduced carrier-scattering [16]. Therefore, Ti-doped ITO films can substitute of Ti^4+^ ions (ionic radius: 0.68 Å) for In^3+^ ions (ionic radius: 0.92 Å), thereby facilitating the fabrication of transparent conductive films with reduced thin-film resistivity. In addition, ITO films doped with Ti exhibit enhanced preferred (222) crystallization, low sheet resistance, and high near-infrared (NIR) transmittance at low post-growth annealing process [17,18,19]. 

In this study, ITO thin films doped with Ti at various growth temperatures were adopted as high-performance TCO films for potential applications in optoelectronic devices. The characteristics of the Ti-doped ITO alloys are thoroughly examined, including their structural, electrical, and optical properties. The resulting high-quality TCO film has a high optical transmittance of 90% at wavelengths of 400–800 nm (VIS light), high carrier concentration of 4.1 × 10^21^ cm^−3^, and lowest resistivity of 1.5 × 10^−4^ Ω/cm at a substrate temperature of 400 °C.

## 2. Experiment

Ti-doped ITO thin films with a thickness of 350 nm were produced using a direct-current magnetron sputtering method, in which a single sputtering target (diameter: 3 inch) was used. The compositional ratio of In_2_O_3_:SnO_2_:TiO_2_ in the sputter target was 94.5%:5%:0.5%. A thin film was deposited on a 2.54 × 2.54 cm^2^ optical EAGLE XG glass substrate (EAGLE XG glass, Corning Incorporated, Corning, NY, USA). The substrate temperature for the thin-film fabrication was varied from room temperature to 500 °C, and the sputtering power was fixed at 100 W. The chamber background pressure and working pressure were 5 × 10^−6^ Torr and 5 mTorr, respectively. Argon (99.99%) was used as the working gas, with a constant flow rate of 30 sccm. 

The optical properties of the thin films were measured using ultraviolet/VIS/NIR spectrophotometer (Perkin Elmer Lambda 750S, PerkinElmer, Waltham, MA, USA) with reference to a glass substrate over a range of optical wavelengths from 400 to 1500 nm. The electrical resistivity, electron mobility and carrier concentration were measured using a Hall measurement system (Lake shore Hall system 7704A, Lake Shore Cryotronics, Westerville, OH, USA) by employing the van der Pauw configuration at room temperature. The crystallite structural properties were examined using X-ray diffraction (XRD, Rigaku TTRAX III, Rigaku Corporation, Tokyo, Japan) with operational voltage and current of 40 kV, 40 mA, and a Cu-Kα (λ = 1.54052 Å) irradiation source in a configuration of grazing incident X-ray diffraction scan with 2θ scanning angle from 20° to 90°. An atomic force microscope (AFM, Veeco Digital Instruments/Dimension 3100, Veeco Instruments Inc., New York, NY, USA) was used in tapping mode to examine the surface morphology and roughness of a 5 × 5 μm^2^ area of the thin films.

## 3. Result and Discussion

**Figure 1:**
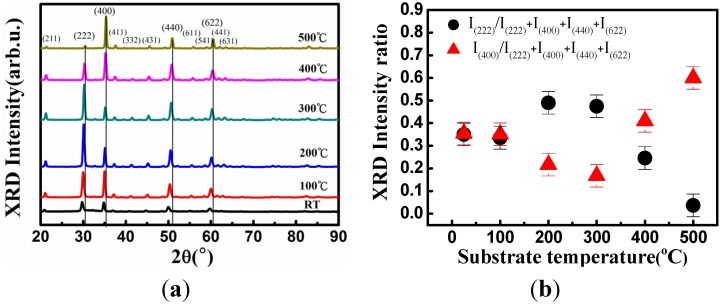
(**a**) X-ray diffraction (XRD) of thin films at various substrate temperatures; (**b**) Diffraction peak intensity ratio of the (222) and (444) orientations, obtained using the peak intensity of the (222) and (400) orientation as the numerator divided by the peak intensity summation of the (222), (400), (440), and (622) orientations at various substrate temperatures. In the XRD measurements, the (222) peak intensity was strongest at 300 °C, and subsequently switched to the (400) preferred orientation as the substrate temperature exceeded 300 °C.

Figure 1a,b show an XRD diagram and the 2θ values of the XRD (222) and (400) peaks of the Ti-doped ITO thin films fabricated at various substrate heating temperatures for faithfully crystallite quality characterization. The four predominant diffraction peaks of the (222), (400), (440) and (622) crystal plane are clearly observable in Figure 1a. Figure 1b depicts the ratio of diffraction peak intensities between the (222) and (400) orientations shown in Figure 1a. The diffraction peak intensity ratio was obtained using the diffraction peak intensity of the (222) and (400) orientation as the numerator divided by the diffraction peak intensity summation of the (222), (400), (440), and (622) orientations. As the temperature of the substrate was increased from room temperature to 200 °C, the (222) orientation diffraction peak value of the thin film increased. As the substrate temperature was further increased from 300 to 500 °C, the (222) orientation diffraction intensity exhibited a rapid decrease, and the lowest diffraction intensity was observed at a substrate temperature of 500 °C. For the (400) orientation diffraction peak, the diffraction intensity began to decrease as the temperature of the substrate was increased from room temperature to 300 °C, yielding the lowest diffraction intensity at 300 °C; it then began to increase as the temperature was increased from 300 to 500 °C until the maximal intensity was observed at 500 °C. This indicated that at low substrate temperatures, the thin film oriented toward the (222) diffraction peak, whereas the preferred orientation of the thin film switched to the (400) crystal orientation at high substrate temperatures. These results indicate that the distance between crystal planes, as well as the internal stress caused by interstitial oxygen atoms in thin films, decreased as the substrate temperature was increased [20,21]. Hence, the diffraction peak values exhibited increased 2θ angles at elevated substrate temperatures.

ITO thin films produced by sputtering growth have been shown to shift toward the (400) orientation at high substrate temperatures. However, an orientation toward (222) was observed in the ITO thin films fabricated using a thermal-evaporation process [20]. The energy (5–10 eV) of particles used for bombardment in the sputtering process was substantially greater than that of thermally evaporated particles, indicating thin-film adatoms require a high amount of energy to grow toward the (400) orientation, which is in contrast to the comparatively lower amount of energy for growing toward the (222) orientation. In the comparative experiment, the thin films grew toward the (222) orientation at low temperatures because the adatoms lacked sufficient energy for the thin films to grow toward the (400) orientation. However, when the temperature reached 500 °C, the adatoms had sufficient energy to migrate along the (400) orientation. Therefore, an increase in XRD (400) orientation diffraction peak intensity was observed in the ITO films with high substrate-temperature growth, as shown in Figure 1a. 

**Figure 2:**
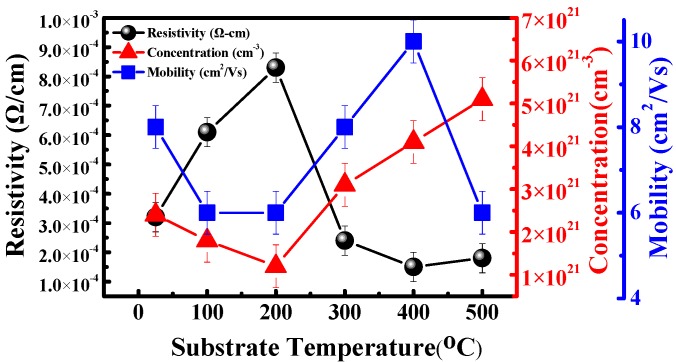
Hall measurement characteristics of electrical resistivity, mobility, and carrier concentration of the Ti-doped indium tin oxide (ITO) thin films deposited at various substrate temperatures, the lowest resistivity is found at the substrate temperature of 400 °C with the highest carrier mobility of 10 cm^2^/Vs.

Figure 2 shows the relationship between the substrate temperature and thin-film electrical properties of the resistivity, carrier concentration, and carrier mobility. The figure indicates that the resistivity of the Ti-doped ITO thin films increased from 3.2 × 10^−4^ to 8.3 × 10^−4^ Ω/cm as the substrate temperature increased from room temperature to 200 °C. As the substrate temperature was increased further, resistivity began to decrease, with the lowest value of 1.5 × 10^−4^ Ω/cm observed at a substrate temperature of 400 °C. When the temperature was increased to 500 °C, the resistivity increased slightly to 1.8 × 10^−4^ Ω/cm. At substrate temperatures below 200 °C, the carrier concentration of the Ti-doped ITO thin films decreased from 2.4 × 10^21^ to 1.2 × 10^21^ cm^−3^ as the substrate temperature was increased from room temperature to 200 °C. At room temperature, the carrier concentration of the Ti-doped ITO thin film was a result of the incomplete oxidation of the Ti-In-Sn alloy. As the substrate temperature was increased to 200 °C, the carrier concentration provided by the incomplete oxidation alloy decreased because of the improved oxidation of the alloys. Thus, the minimum carrier concentration of 1.2 × 10^21^ cm^−3^ was observed at the substrate temperature of 200 °C.

However, when the substrate temperature exceeded 200 °C, the carrier concentration increased as the temperature increased. The increased temperature enhanced the substitution of tin and titanium atoms for indium atoms, causing an increase in carrier concentration in the indium oxide semiconductor. Furthermore, the increased substrate temperature facilitated desorption of interstitial oxygen atoms at the grain boundary, which typically occurs in the thin-film formation, thereby improving the growth of the crystal structure. The improved crystal growth and reduced grain boundary also contributed to the release of the electrons trapped at the grain boundary, which increased the thin-film carrier concentration. As the substrate temperature reached 500 °C, the carrier concentration increased to the maximal value of 5.1 × 10^21^ cm^−3^ [22,23].

The carrier mobility increase from 6 to 10 cm^2^/Vs that occurred as the substrate temperature was increased from 200 to 400 °C was due to the improved crystallite agglomeration and reduced grain boundary [24]. Nevertheless, the carrier mobility was reduced to 6 cm^2^/Vs at the substrate temperature of 500 °C because of the declined crystal quality, as verified in the XRD study.

The resistivity decreased substantially when the substrate temperature was increased beyond 200 °C. As the substrate temperature was increased, the tin ions in the grains were distributed evenly, enabling the thin-film components to achieve an optimal stoichiometric ratio [25]. During high-temperature growth metal ions, such as those in tin and titanium ions, can dissolve into the indium oxide structure through heating energy, thereby substituting the indium ions and releasing free carriers. Furthermore, additional oxygen vacancies generated at high temperatures can provide more free electrons. Therefore, the increased carrier concentration is believed to have contributed to the reduced thin-film resistivity. Low carrier mobility was observed in the range of 6–10 cm^2^/Vs. Because the Ti-doped ITO thin films had high carrier concentrations exceeding 1.0 × 10^21^ cm^−3^, the Coulomb force between ions and electrons as well as the carrier scattering effect were the dominant mechanism in suppressing the carrier mobility.

**Figure 3:**
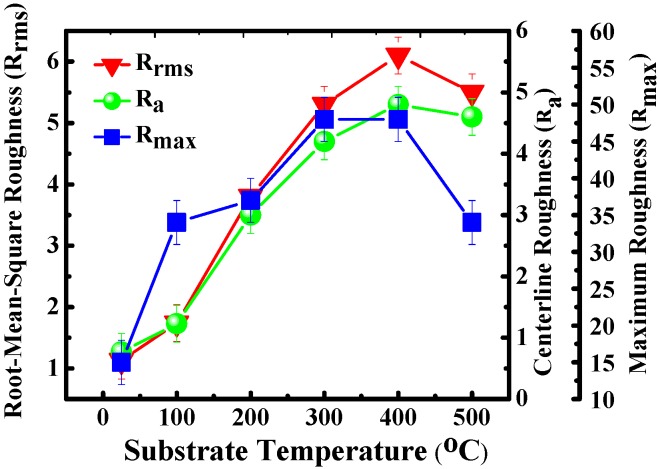
Surface roughness characteristics of the Ti-doped ITO thin films deposited at various substrate temperatures. The roughness of surfaces is represented as the root mean square roughness *R*_rms_, central line roughness *R*_a_, and maximal roughness *R*_max_. The *R*_rms_, *R*_a_ and *R*_max_ were observed increased to the maximal value of 6.1, 4.8 and 48 nm as the substrate temperature was increased to 400 °C.

Figure 3 shows the surface roughness of the thin films, as measured by AFM at various substrate temperatures. The roughness of surfaces is represented as the root mean square roughness *R*_rms_, central line roughness *R*_a_, and maximal roughness *R*_max_. At room temperature, the *R*_rms_, *R*_a_ and *R*_max_ were 1.1, 0.8 and 15 nm, respectively. All the surface roughness values of *R*_rms_, *R*_a_ and *R*_max_ were observed as increasing to their maximal values of 6.1, 4.8 and 48 nm as the substrate temperature was increased to 400 °C. The increased roughness of the surface indicated that the increased mean crystallite size was caused by mutual interaction and agglomeration at high substrate temperatures. However, the *R*_rms_, *R*_a_ and *R*_max_ values decreased slightly to 5.5, 4.6 and 34 nm as the temperature was increased further to 500 °C. Due to the decreased XRD peak intensity and broadened full width at half maximum were observed as the substrate temperature increased to 500 °C, the reduced crystal quality and grain size (calculated using Scherrer’s formula in the XRD study) can be seen as being responsible for the reduced surface roughness. 

**Figure 4:**
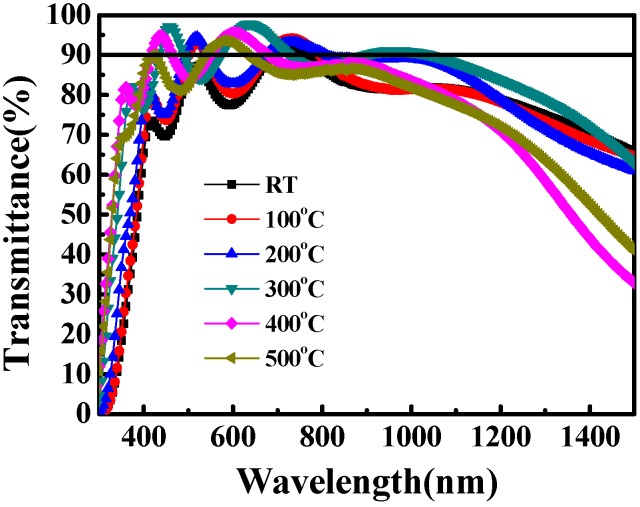
Optical transmittance of Ti-doped ITO thin films prepared at various substrate temperatures. The mean transmittance of 90% at wavelengths of 400–800 nm was achieved by increasing the substrate temperature to 400 °C. RT: room temperature.

Figure 4 shows the mean thin-film transmittance of the Ti-doped ITO thin films at distinct substrate temperatures. The film thickness was fixed at 350 nm. The figure shows that the mean VIS-light (400–800 nm) transmittance of the thin films increased from 83% to 90% as the substrate temperature was increased from room temperature to 400 °C. At 500 °C, the VIS-light transmittance decreased slightly to 87%. The NIR-light area mean transmittance (800–1500 nm) of the thin film also increased as the substrate temperature increased. The NIR-light transmittance of the thin film increased from 77% (at room temperature) to 83% (at 300 °C). However, the NIR transmittance of the thin films decreased to 70% as the substrate temperature was increased further to 400 °C (Table 1). The closed dependence of increased thin-film transmittance with substrate temperature might be correlated with crystal quality and the increased crystallite size [20,24,26]. Since surface adatoms possess high migration energy at high substrate temperatures, the crystallinity increased, as evidenced by the increased XRD (400) orientation intensity and surface roughness observed using the AFM. Therefore, the improved crystal quality, and the reduced grain-boundary scattering, contributed to the increase in optical transmittance as the substrate temperature was increased.

**Table 1:** The visible-light transmittance and sheet resistance of Ti-doped indium tin oxide (ITO) thin films with various substrate temperatures. The figure of merit (Φ_TC_) was used, expressed as Φ_TC_ = *T*^10^/*R*_S_, where *T* is the average transmittance in a visible wavelength region in a wavelength range from 400 to 800 nm, and *R*_S_ is the sheet resistance of the transparent conducting oxide (TCO) structure. NIR: near-infrared.

Item	25 °C	100 °C	200 °C	300 °C	400 °C	500 °C
Visible Light	83%	86%	87%	90%	90%	87%
NIR-light	77%	77%	79%	83%	70%	71%
Sheet resistance (Ω/square)	9.1	17.4	23.7	6.9	4.3	5.1
Figure of merit (×10^−3^ Ω^−1^)	17.1	12.7	10.5	50.5	81.1	48.7

**Figure 5:**
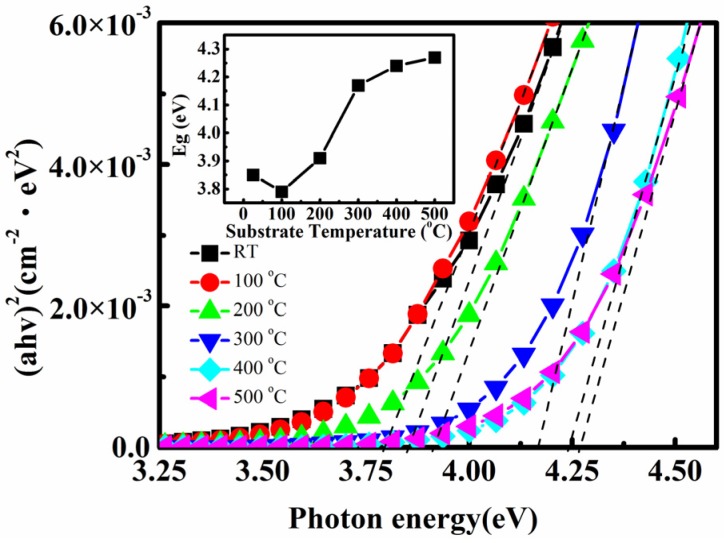
Optical band gap of Ti-doped ITO thin films prepared under various substrate temperatures. The figure indicates optical bandgap the increased from 3.85 to 4.27 eV as the substrate temperature was increased from room temperature to 500 °C, which was primarily attributed to the Burstein-Moss effect.

Figure 5 presents the optical bandgap of the Ti-doped ITO thin films prepared at various substrate temperatures. The results indicate that the optical bandgap increased from 3.85 to 4.27 eV as the substrate temperature was increased from room temperature to 500 °C. The observed blue-shift behavior of the absorption edges at elevated substrate temperatures was primarily attributed to the Burstein-Moss effect (*i.e.*, shifts of the Fermi level caused by the increased concentration of the conduction electrons) [24]. This postulated carrier increment is in agreement with the Hall measurement results shown in Figure 2. Therefore, the Ti-doped ITO films exhibited a short-wavelength absorption edge with a high optical transmission.

The bandgap increase of the Burstein-Moss effect is $\Delta E_{g}^{\text{BM}}$:

(1) $$\Delta E_{g}^{\text{BM}}=\frac{1}{2m_{\text{vc}}^{\text{*}}}{\left(3\pi^{2}n\right)}^{\frac{2}{3}}$$

where *n* is the carrier concentration; and m^*^_vc_ is the effective mass calculated by $\frac{1}{m_{\text{vc}}^{\text{*}}}=\frac{1}{m_{v}^{\text{*}}}+\frac{1}{m_{c}^{\text{*}}}$, in which *m*_v_^*^ and *m*_c_^*^ denote the effective electron mass in the valence and conduction bands, respectively. The equation shows that the increase in carrier concentration and photonic bandgap correlates positively with *n*^2/3^. Thus, the increase in carrier concentration also contributed to the increase of the optical bandgap shown in Figure 5.

To evaluate the performance of Ti-doped ITO in this work, the figure of merit (Φ_TC_) was used, expressed as Φ_TC_ = *T*^10^/*R*_S_, where *T* is the average transmittance in a visible wavelength region in a wavelength range from 400 to 800 nm, and R_S_ is the sheet resistance of the TCO structure using a Hall measurement system by employing the van der Pauw configuration at room temperature [27,28]. Table 1 shows the Φ_TC_ calculation results of Ti-doped ITO, which indicates the highest value of 81.1 × 10^−3^ Ω^−1^ at a substrate temperature of 400 °C. Therefore, a substrate temperature of 400 °C for Ti-doped ITO thin films is suggested for high-performance TCO applications.

## 4. Conclusions

In conclusion, this study evaluated the effects of substrate temperature on Ti-doped ITO thin-film growth. In the XRD measurements, the (222) peak intensity was strongest at 300 °C, and subsequently switched to the (400)-preferred orientation as the substrate temperature exceeded 300 °C. The Ti-doped ITO thin films exhibited superior optoelectronic characteristics at a substrate temperature of 400 °C. The thin-film resistivity demonstrated advantageous resistivity of 1.5 × 10^−4^ Ω/cm, carrier concentration of 4.1 × 10^21^ cm^−3^, and carrier mobility of 10 cm^2^/Vs. The Ti-doped ITO thin-film surface roughness exhibited a pronounced increase as the substrate temperature was increased. The mean transmittance of 90% at wavelengths of 400–800 nm was achieved by incorporating Ti into the ITO thin films with a substrate temperature of 400 °C. The superior carrier concentration of the Ti-doped ITO alloys (>10^21^ cm^−3^) with a high figure of merit (81.1 × 10^−3^ Ω^−1^) demonstrate the pronounced contribution of using Ti as a dopant, indicating high suitability application in optoelectronic devices. Therefore, increasing the substrate temperature can substantially facilitate thin-film growth and improve the optoelectronic properties of Ti-doped ITO TCO thin film.
